# Treatment of the Cervical Border

**Published:** 1899-01

**Authors:** Stafford G. Perry

**Affiliations:** New York.


ARTICLE VII.

### TREATMENT OF THE CERVICAL BORDER.

DR. STAFFORD G. PERRY, NEW YORK.

In every approximal cavity, in beginning the filling, the end of the plugger forces the gold squarely against the cervical border, and as the instrument is applied at right angles, the gold can be most easily and accurately adapted to the tooth with least force.

As the curve of the cavity is reached on either the lingual or the buccal aspect, the directness of application of force is lost, and this doubtless accounts for the fact that so many failures commence at this point on either the buccal or lingual sides of the tooth. By the time the cavity is about one-third filled and until finished the instrument is packing along the sides of the walls, instead of squarely against them. This natural position is the redeeming feature of the cervical border, and has made it possible for many botch operators to make fillings that were successful at this point. I am under the impression that some operators, appreciating this fact of the square application of the instrument along this border, use thicker mats or masses of gold than they are able to condense thoroughly, even though considerable mallet force is applied. I have repeatedly seen fillings that were solid and fine in other parts, and yet along this border were soft and imperfect. In fact, I think we are all inclined to work too rapidly in packing the gold here, and also that we use the gold in too large masses.

For this purpose I have devised and used for many years plugger-points which are adapted to bring pressure on the edge of the cavity, and which, being made flat on the side which rests against the matrix, insures a very perfect adaptation of the gold. These are made for the cavities on the anterior and posterior sides of the teeth respectively. It is not easy to describe their exact shapes, but I think they will be appreciated when seen.

There has been much said for and against a loose-fitting matrix—this is, one that stands off from the tooth somewhat, so that the gold can be packed a little beyond the cavity, thereby insuring a better adaptation at the edge. There is some reason in this, and yet it may be an open question if the time spent in finishing such a filling were given to packing the gold in smaller pieces, and with the instrument just described, against a close-fitting matrix, if an equally good edge would be secured.

"The peculiar "rake" of these points makes it possible to make a perfect edge if we will be patient and careful. Of course, it is assumed that the matrix is bulged to conform to the natural shape of the tooth. If an equally good edge can be made with a close-fitting matrix, and as quickly, when the whole operation is considered, there is a gain in saving the patient the disagreeable task of trimming the gold, which generally results in disturbance of the gum from the use of files or chisels and the dreaded sand-paper strips. In the early years of the matrix I was shy of it, fearing the making of bad margins; but since having my own plugging instruments, my use of the matrix has increased, while my dependence on the separator has lessened.

A word more now, even at the risk of repetition, in reference to the cervical border. If gentleness and close care in filling characterize our method, and some soft or semi-soft gold is to be our main reliance, then I think we need not cut so radically in the preparation of this border.

Of course, this will be for the purpose of avoiding the encroachment on the thin enamel, and to save the disturbance of the gum, as well as to make the operation easier for both patient and operator, as before stated. If it is said we should not stop short of a radical operation, whatever effort to the operator or discomfort to the patient, if it only betters an operation, then I reply, "Is it a better operation?" When all the conditions are considered, I answer, "I think not." Nature is a safe guide, and I think we work well when we strive to leave a natural condition of the gum and of the teeth at the cervical margin.

It is perhaps a little curious that after the lapse of a quarter of a century I should have to ask for moderation in the application of such a method. But the confidence of youth is beyond belief.

I now come naturally to the consideration of plastics in their relations to this border. And here let me tell you frankly that, for my appearance before you, I selected this subject partly for one particular purpose—namely, to emphasize the danger of using oxyphosphate at this border without preceding it by some insoluble and indestructible substance. Oxyphosphate of zinc has been one of the most valuable acquisitions in dentistry, and it has been one of the most dangerous. I shall feel well repaid for whatever effort it has cost me if I can only succeed in making a few members of the profession appreciate a little more fully the danger of using oxyphosphate along the cervical border. It may be that it is not generally used in this place, and that my anxiety is unnecessary; but it is my observation that it is generally so used here by most operators in this country and by those who practice abroad, and without being preceded by any other indestructible substance. I am sure there is not one of us who would not to-day be crippled in our practice by the withdrawal of oxyphosphate, and at the same time I believe there are not many of us who fully appreciate its danger.

In the beginning I said that the cervical border was the most inaccessible and most vulnerable of any cavity border in the mouth. If against this border you will use a substance so liable to chemical disintegration as oxyphosphate, you will have intensified the conditions of danger, and though you have delayed the evil hour of the tooth's destruction, you have established a set of conditions that in time will bring it about with almost scientific certainty.

It would not be easy to estimate the number of pulps that have become exposed by the treacherous washing out of this material at the cervical border, thus allowing decay to go on quietly, without giving any intimation of its existence till too late. And the same may be said of the copper amalgam which was so much used a few years ago, and which dissolved at the cervical border in nearly the same manner. It left a dirty trail, and the memory of it is a nightmare. But we are not concerned with it in this paper, and let us hope it is a thing of the past.—*International.*

